# Supplementary material for: Cellular and Molecular Basis of Environment-Induced Color Change in a Tree Frog
Source: Animals (Basel). 2024 Dec 1;14(23):3472. doi: 10.3390/ani14233472 (PMC11640764; doi:10.3390/ani14233472)
Supplement: Supplementary file 1 [file animals-14-03472-s001.zip › animals-3299412-supplementary.pdf]

Supplementary Materials:

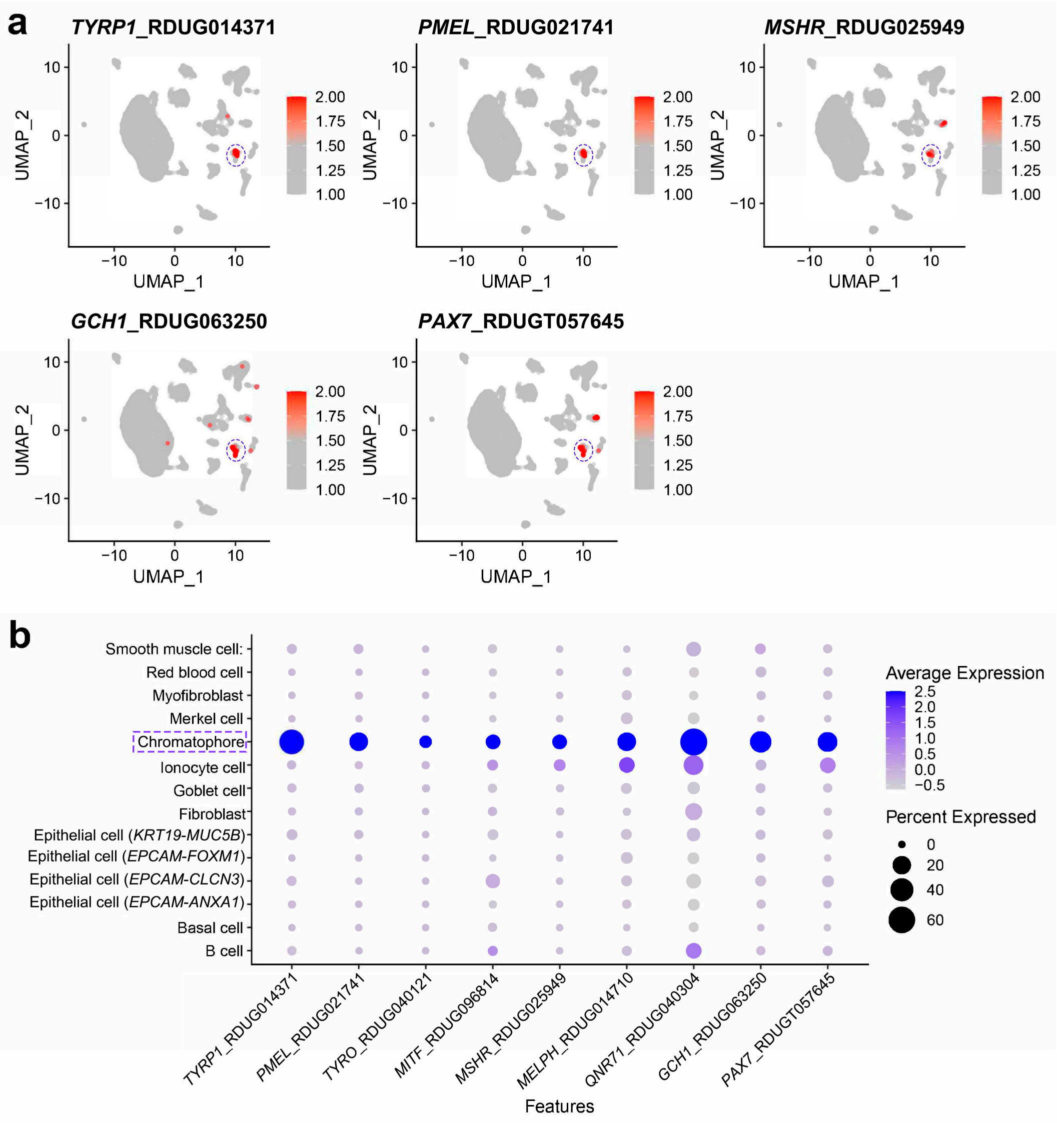

Figure S1 Validation of chromatophores. **(a)** UMAP feature plots presenting the cell-specific expression of marker genes of chromatophore. **(b)** Dot plot presenting the cell-specific expression of marker genes of chromatophore.

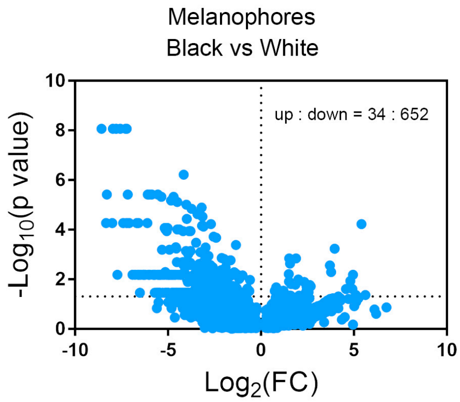

Figure S2 Volcano plot presenting the gene expression difference in melanocyte of individuals form different background colors.
